# Association between anemia and quality of life in a population sample of individuals with chronic obstructive pulmonary disease

**DOI:** 10.1186/1471-2466-6-23

**Published:** 2006-09-05

**Authors:** Gokul Krishnan, Brydon J Grant, Paola C Muti, Archana Mishra, Heather M Ochs-Balcom, Jo L Freudenheim, Maurizio Trevisan, Holger J Schünemann

**Affiliations:** 1 Department of Medicine, School of Medicine and Biomedical Sciences, University at Buffalo, Buffalo, US; 2 Department of Social and Preventive Medicine, School of Public Health and Health Professions, University at Buffalo, Buffalo, US; 3 Section of Pulmonary, Critical Care, and Sleep Medicine, Veterans Administration Medical Center, Buffalo, US; 4 Department of Epidemiology, Italian National Cancer Institute Regina Elena, Rome, Italy; 5 Clinical Research and INFORMAtion Translation Unit (INFORMA), Italian National Cancer Institute Regina Elena, Rome, Italy

## Abstract

**Background:**

Several studies investigated the association of anemia with health related quality of life (HRQL) in patients with chronic disease. However, there is little evidence regarding the association of anemia with HRQL in patients with chronic obstructive pulmonary disease (COPD).

**Methods:**

This is a post-hoc analysis of a study which enrolled a population of adults aged 35–79 randomly selected from residents of Erie and Niagara Counties, NY, between 1996 and 2000. In addition to demographic information and physical measurements, we obtained spirometry data and hemoglobin levels. We used modified Global Initiative for Chronic Obstructive Lung Disease (GOLD) criteria to define COPD, and World Health Organization (WHO) criteria to define anemia. To assess HRQL we used the Short Form-36 (SF-36) to assess physical functioning (PF), physical component summary (PCS) measures and mental component summary (MCS) measures.

**Results:**

In the entire study population (n = 2704), respondents with anemia had lower scores on the physical functioning domain [45.4 (SD10.9) vs. 49.2 (SD 9.1); p < 0.0001]. Among patients with COPD (n = 495) the PF scores (39.9 vs. 45.4) and the PCS (41.9 vs. 45.9) were significantly lower in individuals with anemia compared to those without. In multiple regression analysis, the association between hemoglobin and PCS was positive (regression coefficient 0.02, p = 0.003). There was no significant association of hemoglobin with PF scores or the mental component summary measure after adjusting for covariates in patients with COPD.

**Conclusion:**

In patients with moderate to very severe COPD anemia may be associated with worse HRQL. However, co-morbidities may explain part or all of this association in these patients.

## Background

Health related quality of life (HRQL) is an important clinical outcome for patients with advanced lung disease and their health care providers [1]. This outcome measure helps differentiate between individuals with varying severity of lung disease and allows to evaluate how much impact a specific intervention has on patients' lives.

Fatigue is a frequent symptom in patients with chronic obstructive pulmonary disease (COPD). Nearly half of the patients with COPD report fatigue every day with negative impact on cognitive, physical, and psychosocial functioning [2]. Thus, fatigue adversely affects HRQL. Fatigue is the primary symptom of anemia [3]. Dyspnea is another manifestation of anemia because of reduced oxygen carrying capacity of the blood. Thus, if patients with COPD suffer from anemia, then fatigue and dyspnea may be worse. Treatment of anemia, therefore, could have favorable effects on fatigue and dyspnea, and, in turn, improve HRQL. In fact, anemia is one of the most treatable cause of fatigue in general [4].

HRQL and anemia have been extensively studied in patients with other morbidities including renal failure and cancer. For example, in patients with end-stage renal diseases investigators showed a strong association between HRQL, hospitalizations, and survival in those with higher hemoglobin levels [5]. Some, but not all studies suggest that HRQL may improve in cancer patients treated with erythropoietin [6-9].

There is limited published evidence about the association of anemia with HRQL in individuals with COPD. We identified three studies that evaluated the prevalence of anemia in patients with COPD. In one study the prevalence of anemia was 36% in COPD patients randomly selected from an outpatient clinic [10]. In another study enrolling 58 patients the prevalence was 48% [11]. The latter study also found a significant correlation between severity of anemia and severity of COPD [11]. A recent study evaluating anemia and inflammation in COPD patients found a 13% prevalence of anemia in patients with COPD [12].

Anemia may be under-diagnosed and it may have adverse effects on fatigue and, thus, HRQL in patients with COPD. If this were the case, treatment of anemia may improve both symptoms and HRQL in this patient population. Therefore, we evaluated whether anemia or hemoglobin levels are associated with HRQL in individuals with COPD randomly selected from the general population.

## Methods

### Study population

This is a post-hoc analysis of a study which enrolled participants from a general population sample from Erie and Niagara Counties, New York, as previously described [13]. In brief, we randomly selected residents of Eric and Niagara counties age 35 to 64 yr from the New York State Department of Motor Vehicles records. For participants aged 65 to 79 yr we used the rolls of the Health Care Finance Association. We assigned a computer-generated random number to each person on the complete lists of all potential participants supplied by the New York State Department of Motor Vehicles and the Health Care Finance Association. We then sorted potential participants with ascending numbers according to their randomly assigned number and they were then contacted in sequential fashion. We mailed introductory letters to potential interviewees before the interviewers' telephone calls. Participants receive up to 12 callbacks. We recruited a total of 4065 subjects from 1996 to 2001. The study was reviewed by the Institutional Review Board of SUNY at Buffalo. All participants signed an informed consent at the time of enrollment.

From the present analysis we excluded participants for the following reasons: missing pulmonary function tests (n = 771); missing blood determination of hemoglobin (n = 100); missing scores on the HRQL measures (n = 143); pulmonary function tests were unacceptable or not reproducible (n = 207); race other than Caucasian or African-American (n = 35); missing information on height, smoking status, pack-years of smoking cigarettes, and education (n = 41); and missing information on co-morbidity data (liver cirrhosis, myocardial infarction, renal diseases or diabetes) (n = 64). Complete data were available for 2704 participants, of whom 495 were classified as having moderate to very severe COPD as described below.

### Interview

The examination included both an in-person interview that addressed a number of life-style habits, including questions on the duration and intensity of lifetime cigarette smoking, and a self administered questionnaire. The questionnaire included questions on education, medical history, and vitamin supplement use. For this study we used the Short Form-36 (SF-36) which SF-36 is the most widely used generic instrument for measuring HRQL [14].

We used co-morbidity data from the questionnaire for multivariate and multivariable analyses. We asked participants whether they were told by a physician or other medical personnel that they have or have had a particular condition. For these analyses, we considered liver cirrhosis, diabetes, myocardial infarction, and renal diseases as covariates. We did not include patients with cancer in our analysis. Before the end of the interview, trained personnel reviewed all answers in the questionnaire and participants clarified any uncertain aspects.

### Pulmonary function tests

Trained personnel performed spirometry between 6:30 and 9:30AM following the American Thoracic Society (ATS) guidelines on the Standardization of Spirometry, 1994 update [13,15]. We calculated forced Expiratory Volume in one second (FEV_1_) and Forced Vital Capacity (FVC) prediction equations for men and women separately with values obtained from the included participants who were lifelong nonsmokers. We used a dummy variable for race (Caucasian = 0, African-American = 1) and included 388 men and 538 women who never smoked and did not report a history of chronic lung disease.

We obtained the following equations for men:

Predicted FEV_1_= -1.38165 - 0.02993 × age (years) + 3.82787 × height (m) - 0.42160 × race

Predicted FVC = -3.23510 - 0.03333 × age (years) + 5.56883 × height (m) - 0.51062 × race

For women we obtained the following equations:

Predicted FEV_1_= -0.00438 - 0.02673 × age (years) + 2.57782 × height (m) - 0.33534 × race

Predicted FVC = -0.94723 - 0.02813 × age (years) + 3.64983 × height (m) - 0.52404 × race

We then used the predicted FEV_1 _and FVC to calculate percent-predicted for each of these parameters. We categorized the participants into stages according to the Global Initiative for Chronic Obstructive Lung Disease (GOLD) criteria [16]. The stages of COPD in our study are based on pre-bronchodilator spirometry parameters including FEV_1_/FVC and FEV_1_. We did not use symptoms to define stages because we did not assess symptoms according to GOLD criteria and, hence categorized patients into stages 2 – 4. Of the 2704 participants, 2206 were in Stage 0, 3 in Stage 1, 434 in Stage 2, 55 in Stage 3 and 6 in Stage 4. So by combining Stages 2–4 we had 495 participants with moderate to very-severe COPD.

### Blood determinations

We determined anemia based on measurement of hemoglobin from a fasting blood sample drawn between 7:30 and 9:30 AM. Trained personnel processed the samples within 30 min, frozen at -80°C, and analyzed samples in batches. An automated differential cell blood count was completed using a Coulter Counter (Beckman Coulter, Inc., Fullerton, CA).

### Anthropometry

The personnel followed a detailed protocol to measure body weight using a balance beam scale (Detecto, Inc., Webb City, MO) and height using a standardized scales (Perspective Enterprises, Kalamazoo, MI).

### Health related quality of life (HRQL) indicators

The SF-36 contains 36 items contributing to eight domains measuring physical function (10 items), role limitations-physical (4 items), bodily pain (2 items), vitality (4 items), general health perceptions (5 items), role limitations-emotional (3 items), social function (2 items), and mental health (5 items). These eight domains are used to calculate the two component summary measures: Physical and Mental. Higher scores on any of the domains or summary scores indicate that the participant has better HRQL. The validity of the 8 domains and the 2 summary measures of the SF-36 has been widely investigated for the general population and for a wide variety of patient groups [14,17].

A priori, we determined that we would use three SF-36 measures as the main outcomes for our analysis: physical functioning (PF), physical component summary (PCS) measures and mental component summary (MCS) measures.

### Statistical analysis

We used SAS^® ^statistical software V9 (Copyright (c) 2002 by SAS Institute Inc., Cary, NC, USA) for the analyses and calculated individual scale scores of the SF-36 using published algorithms [18]. We computed raw SF-36 scores and then transformed these scores to the commonly used 0 – 100 normed metric [18]. We determined a priori to focus our analysis on the physical functioning domain and the two SF-36 summary scores.

#### Definitions

We defined anemia using hemoglobin levels of less than 12 g/dl of hemoglobin in women and less than 13 mg/dl in men using the WHO criteria [19]. Never-smokers were defined as those who had smoked less than 100 cigarettes during their entire lifetime and we derived pack-years of smoking exposure from total years of smoking multiplied by the number of cigarettes per day divided by 20.

#### Covariates

We considered the following covariates in the analyses: education (years of education used as a continuous variable), gender, race, age, cumulative tobacco smoke exposure (pack-years of smoking) and the co-morbidities liver cirrhosis, diabetes, myocardial infarction, and renal diseases as described earlier. We used education as an indicator of socioeconomic status. We compared the demographic data for participants with anemia and those without anemia using the unpaired t-test and chi-square.

To exclude outliers in the data we examined studentized residuals in the regression procedure, and we excluded observations from our analysis if the residual was 2.5 or higher. For physical functioning there were 11 outliers, for PCS there were 6 outliers and for MCS there were 14 outliers. Unadjusted mean scores of the outcome variables were then generated for the two groups among participants with COPD using a General Linear Model (GLM). Applying Analysis of Covariance (ANACOVA) we calculated mean scores after adjusting for the aforementioned covariates.

#### Main analysis

Based on quartile analysis by gender we generated scores for each of the three outcome variables (physical functioning and the two SF-36 summary scores) across four levels of anemia to investigate trends. We then performed bivariate analyses of the HRQL measures and the independent variables as well as the covariates. To account for lack of linearity in our outcome variables based on trend analysis we used log transformation of each of the outcome variables in our regression analysis. For physical functioning total pack years of smoking, history of myocardial infarction and renal diseases were not included because they were not statistically significant in the bivariate analyses. For PCS we did not include the following variables in our final model because they were not statistically significant: pack years of smoking, gender, history of myocardial infarction and renal diseases. We used *backward elimination *multiple linear regression analysis to analyze the relationship between anemia and the three outcome variables in participants with COPD after adjustment for other covariates in the model. In all analyses, we defined significance as p < 0.05.

## Results

**Figure 1:**
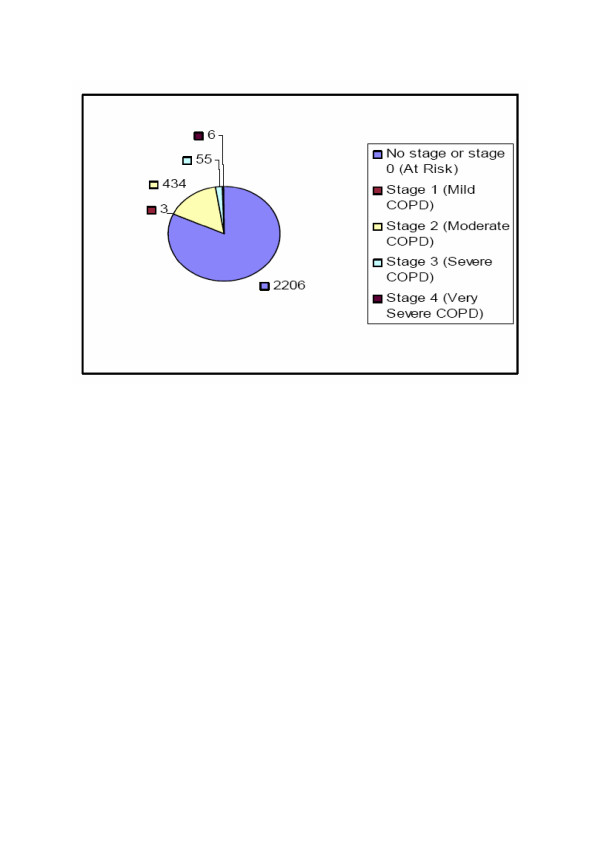
Distribution of GOLD stages in the study population (pre-bronchodilator spirometry).

We analyzed the demographic characteristics and the mean scores for all eligible participants by anemia status and in the entire study group (N = 2704) (Data not shown). The prevalence of anemia in the included population was 5.3%. There was no significant difference in mean age, gender distribution, and history of myocardial infarction between the two groups (anemia vs. no anemia). The mean physical functioning score and the PCS were significantly lower in participants with anemia. There was no significant difference between the two groups in MCS score, total pack years of smoking, and FEV_1_. The number of African-American participants was significantly higher in the group with anemia. In regards to other co-morbidities the group with anemia appeared to have significantly higher prevalence of diabetes, renal diseases, and liver cirrhosis. Of all participants, 495 were classified as moderate to severe COPD. Characteristics of excluded participants were similar to those mentioned above (mean age 59.4 years (SD 12.4), 46.7% male and 88.6% Caucasian). Figure 1 shows the distribution of participants by GOLD stages in a pie-chart (note that we did not specifically assess these stages because we completed the evaluation before publication of the GOLD criteria).

Table 1 shows the demographic data for the group of participants with COPD. The prevalence of anemia was 7.5%. There was no difference between those with and without anemia in their average age, gender distribution, history of renal diseases, history of myocardial infarction, years of education, and FEV_1_. In those with anemia there was a significantly higher percentage of participants with diabetes.

Unadjusted analysis showed significantly lower physical functioning (p = 0.002) and PCS (p = 0.02) scores but not MCS scores in COPD patients with anemia (Table 2). When we examined the data after stratifying by gender we found that the PF and PCS scores are significantly different between the two groups only for females (36.3 vs. 44.6 for PF and 39.2 vs. 45.5 for PCS). There is no significant difference between the scores for males. However, after adjusting for covariates, both the physical functioning scores and the PCS were not statistically significantly related to anemia (Table 3).

Table 4 shows results of multivariable regression analysis for physical functioning. After adjusting for covariates there was no significant association between hemoglobin and the log-transformed physical functioning score (p value: 0.35). For PCS we observed a significant association between hemoglobin and log-transformed PCS (p = 0.003, Table 5). There was also no significant association between MCS and hemoglobin after adjusting for covariates.

## Discussion

We analyzed the association between anemia and HRQL in individuals with COPD in a population-based sample. In these individuals, HRQL (PCS) assessed with the SF-36 was negatively and significantly associated with hemoglobin levels even after adjustment for co-variates. Although we found no statistically significant associations with the MCS or the PF domain of the SF-36 after adjustment for covariates, these results suggest that low hemoglobin levels are associated with impaired HRQL in individuals with moderate or severe COPD.

Our study has several strengths. To our knowledge there is no information from population based studies on the association between anemia and HRQL in individuals with COPD and, thus, this study provides new information. In addition, we collected detailed information on covariates and confounders that allowed for adjusted analyses.

The study also has some limitations. First, we neither evaluated respiratory symptoms nor measured post-bronchodilator FEV_1_. Thus, we could not apply the original GOLD criteria published after initiation of this study. However, we used a transparent modified approach of the GOLD criteria to define COPD that could improve comparison with future studies. Many epidemiological studies will be limited in that they do not collect post-bronchodilator spirometry. Second, readers should not infer causation from the cross-sectional association of HRQL impairment and anemia in individuals with COPD, because it is possible that residual confounding by comorbidities is responsible for the observed association.

Evidence for an association between HRQL and hemoglobin levels is limited to two studies published in abstract form [10,11]. The study by Stanbrook et al. explored the prevalence of anemia in COPD patients [10]. The authors randomly selected patients from outpatients of a pulmonary subspecialty clinic and from a pulmonary rehabilitation program. Anemia was defined as <14 g/dl for men and <12 g/dl for women. Prevalence of anemia was 36%. Park et. al performed another retrospective study to determine the prevalence of anemia in COPD patients [11]. Data from 58 COPD patients recruited from a pulmonary clinic showed anemia to be present in 48%. The prevalence of anemia in our study was considerably less than that observed in these clinic-based studies (7.5% vs. 36% and 48%). A likely explanation for this difference is that individuals from the general population participated in our study rather than patients in tertiary clinics who may be sicker than participants in a general population study. Hence it is possible that stronger associations between anemia and HRQL might be observed among patients with severe COPD drawn from a tertiary lung clinic.

We did not specifically assess hemoglobinopathy in this study despite of race being an important variable. To inform this issue we evaluated the Mean Corpuscular Volume (MCV) which was available for all included subjects. There was no statistical difference in mean MCV or mean hemoglobin between African-Americans and Caucasians in either the overall population or in those with moderate to very severe COPD (Data not shown). Although this latter evaluation does not address the issue directly it lends some support to the notion that hemoglobinopathies did not play an important role or they were similarly distributed in these two races.

The difference between males and females deserve some consideration. We are unable to explain why the difference in HRQL scores was greater in women compared to men. One possible conclusion is that females with anemia perceive greater impairment of their HRQL compared to males as has been seen in various studies and reviews [20-23]. There has been only one systematic review which has addressed this issue specifically and it suggests that women may report symptoms and worse HRQL compared to men [24]. However, one should also note that our results come from an explorative post-hoc analyses and it is possible that they represent a false positive result.

Although we included and adjusted for some comorbidities, we did not account for some of the disease processes which can influence HRQL such as congestive heart failure and neuromuscular disorders. Medications such as Angiotensin Converting Enzyme inhibitors have been associated with anemia [25], but we did not adjust for this possible confounder in our analysis, because the evidence is limited and the cause effect relation not established.

The association between HRQL and anemia as a dichotomous variable was weaker (Table 2) than the association between HRQL and hemoglobin levels (Table 4). The latter is a result of the increased power in the analysis using the continuous variable compared to the binary variable of presence of anemia. However, adjustment for comorbidities weakened the relation between hemoglobin levels and HRQL suggesting that comorbidities play a role in the association between anemia and HRQL impairment. Using the PCS of the SF-36 reduces random error and increases the importance attributed to impairment of HRQL related to physical impairment. The latter is due to the weighing of all 8 domain scores on the SF-36 that make up the PCS. Although we speculated that low hemoglobin levels may contribute to impairment of mental function assessed with the MCS of the SF-36, we did not observe an association between the two variables. Thus, anemia may primarily affect physical functioning as a whole and have less impact on any emotional consequences of fatigue and dyspnea.

Although there are several disease specific HRQL questionnaires for individuals with COPD [26-28], our study utilized a generic instrument, the SF-36. The SF-36 has shown good discriminative properties; that is the ability to differentiate between individuals with better or worse HRQL cross-sectionally [17] Our study aimed at the general population of which we did not – a priori – know who had COPD. For our analysis, discriminative properties were of primary concern and therefore, the SF-36 represented an appropriate measurement tool [29].

What implications do our findings have for clinical practice? We have not established a causal relation between lower hemoglobin levels and HRQL. Thus, no treatment recommendations follow from our results. However, we encourage practitioners to explore underlying etiologies for anemia as this may have implications for HRQL in an individual with COPD.

## Conclusion

In conclusion, our analyses provide evidence for a negative association between HRQL and hemoglobin levels in individuals with moderate -to-severe COPD. Co-morbidities appear to explain part of the observed association for PF and MCS but not for PCS. If, in fact, there is a causal relationship, there could be important implications for treatment of anemia to improve HRQL in individuals with COPD. However, additional observational studies are required to confirm our findings before intervention studies can be advocated in individuals with low hemoglobin levels and COPD.

## Abbreviations

1. HRQL: Health Related Quality of Life

2. COPD: Chronic Obstructive Pulmonary Disease

3. GOLD: Global Initiative for Chronic Obstructive Lung Disease

4. WHO: World Health Organization

5. SF-36: Short Form-36

6. PF: Physical Functioning domain of SF-36

7. PCS: Physical Component Summary Measure of SF-36

8. MCS: Mental Component Summary Measure of SF-36

9. ATS: American Thoracic Society

10. FEV_1_: Forced Expiratory Volume in One second

11. FVC: Forced Vital Capacity

12. ANACOVA: Analysis of Covariance

GLM: General Linear Model

## Competing interests

Holger J Schünemann has deposited honoraria and unrestricted research funds received from Amgen, Inc., in 2003 into a Department of Medicine, University at Buffalo, research account as per CLARITY Research group policy.

## Authors' contributions

GK carried out data analysis, data interpretation and drafting of the manuscript. BJBG carried out data interpretation and statistical supervision. PM carried out design, obtaining funding, data interpretation. AM carried out data interpretation. HOB participated in data acquisition, data analysis, data interpretation. JF carried out design, obtaining funding and data interpretation. MT carried out study desgin, obtaining funding and data interpretation. HJS carried out study design, funding, analysis, data interpretation, manuscript preparation. All authors read and approved the manuscript.

## Pre-publication history

The pre-publication history for this paper can be accessed here:



## Figures and Tables

**Table 1:** Demographic Characteristics of Subjects with COPD by Anemia Status (n = 495)

**Characteristic**	**Anemia (n= 37)**	**No Anemia (n= 458)**	**Total (n= 495)**	**P-Value for Difference**
**Age (Means, SD)**	65.05 (10.45)	64.07 (9.94)	64.15 (9.97)	0.57
**HGB (Means, SD)**	11.51 (1.05)	14.63 (1.11)	14.40 (1.38)	<0.0001
**Men (n, %)**	19 (51.35)	277 (60.48)	296 (54.80)	0.28
**Years of Education (Mean, SD)**	12.46 (2.59)	12.84 (2.43)	12.81 (2.44)	0.36
**Race (n, %)**				<0.0001
Caucasian	25 (67.57)	426 (93.01)	451 (91.11)	
African American	12 (32.43)	32 (6.99)	44 (8.89)	
				
**Myocardial Infarction (n, %)**	3 (8.11)	53 (11.57)	56 (11.31)	0.52
**Renal Disease (n, %)**	2 (5.41)	8 (1.75)	10 (2.02)	0.13
**Diabetes (n, %)**	14 (37.84)	68 (14.85)	82 (16.57)	0.0003
**Smoking Status (n, %)**				0.09
Never Smoked	13 (35.14)	107 (23.36)	120 (24.24)	
Current Smoker	5 (13.51)	129 (28.17)	134 (27.07)	
Former Smoker	19 (51.35)	222 (48.47)	241 (48.69)	
				
**Total Pack Years Smoked**	18.65 (22.67)	28.33 (26.71)	27.60 (26.53)	0.03
**FEV_1_, % Predicted**	68.43 (8.23)	66.04 (12.69)	66.22 (12.42)	0.26

**Table 2:** Summary of Unadjusted Hemoglobin Analysis in Subjects with COPD (Stage 2, 3, 4 of GOLD Criteria)

**Outcome Variables**	**Unadjusted Group Means (Std. Error)**		**P-Value**
		
	**Anemia**	**No Anemia**	
**Physical Functioning Scores**	(n = 37) 39.98 (1.68)	(n = 447) 45.36 (0.48)	0.002
**PCS**	(n = 37) 41.95 (1.54)	(n = 452) 45.86 (0.44)	0.02
**MCS**	(n = 37) 49.83 (1.06)	(n = 444) 49.07 (0.30)	0.49

**Table 3:** Summary of Adjusted Hemoglobin Analysis in Subjects with COPD (adjusted for age, gender, years of education, pack years of smoking, history of myocardial infarction, renal diseases or diabetes and race)

**Outcome Variables**	**Adjusted Group Means (Std Error)**		**P-Value**
		
	**Anemia**	**No Anemia**	
**Physical Functioning Scores**	(n = 37) 39.26 (2.27)	(n = 447) 42.15 (1.90)	0.095
**PCS**	(n = 37) 40.62 (2.08)	(n = 452) 43.05 (1.70)	0.14
**MCS**	(n = 37) 51.81 (1.48)	(n = 444) 51.34 (1.23)	0.68

**Table 4:** Multiple Linear Regression Analysis for Association Between Hemoglobin Level and Physical Functioning Scale (*log transformed*) after Adjusting for Baseline Characteristics (Following variables were removed because of lack of significant association in bivariate analysis: pack years of smoking, history of myocardial infarction and renal diseases)

**Variable (n = 484)**	**Regression Coefficient**		**P Value**	**R**^**2**^
			
	**Beta**	**Std Error**		
**Hemoglobin**	0.01	0.01	0.35	0.1214
**Age**	-0.003	0.001	0.002	
**Gender (Female ref)**	0.06	0.03	0.04	
**Race (Caucasian ref)**	-0.11	0.04	0.01	
**Education**	0.02	0.005	0.002	
**Diabetes**	-0.13	0.03	<0.0001	

**Table 5:** Multiple Linear Regression Analysis for Association Between Hemoglobin Level and Physical Component Summary Measure (*log transformed*) after Adjusting for Baseline Characteristics (Following variables were removed because of lack of significant association in bivariate analysis: pack years of smoking, history of myocardial infarction and renal diseases, and gender)

Variable* (n = 489)	**Regression Coefficient**		**P Value**	**R**^**2**^
			
	**Beta**	**Std Error**		
**Hemoglobin**	0.02	0.01	0.003	0.0850
**Age**	-0.002	0.001	0.04	
**Education**	0.02	0.004	<0.0001	
**Diabetes**	-0.07	0.03	0.02	

*Race was eliminated in model selection
